# Role of the Laboratory in Ensuring Global Access to ARV Treatment for HIV-Infected Children: Consensus Statement on the Performance of Laboratory Assays for Early Infant Diagnosis

**DOI:** 10.2174/1874613600802010017

**Published:** 2008-03-10

**Authors:** W Stevens, G Sherman1, R Downing, L.M Parsons, C.-Y Ou, S Crowley, G.M Gershy-Damet, K Fransen, M Bulterys, L Lu, J Homsy, T Finkbeiner, J.N Nkengasong

**Affiliations:** 1 University of the Witwatersrand and National Health Laboratory Service, Johannesburg, South Africa, Global AIDS Program; 2 U.S. Centers for Disease Control and Prevention, Kampala, Uganda, Global AIDS Program,; 3 U.S. Centers for Disease Control and Prevention, Atlanta, Georgia, USA, Global AIDS Program,; 4 World Health Organization, Geneva, Switzerland,; 5 World Health Organization, Harare, Zimbabwe; 6 Institute of Tropical Medicine, Antwerp, Belgium; 7 Global AIDS Program, U.S. Centers for Disease Control and Prevention, Lusaka, Zambia

**Keywords:** HIV infection, early infant diagnosis, laboratory assays, HIV DNA PCR

## Abstract

A two day meeting hosted by the World Health Organization (WHO) and the U.S. Centers for Disease Control and Prevention (CDC) was held in May 2006 in Entebbe, Uganda to review the laboratory performance of virologic molecular methods, particularly the Roche Amplicor DNA PCR version 1.5 assay, in the diagnosis of HIV-1 infection in infants. The meeting was attended by approximately 60 participants from 17 countries. Data on the performance and limitations of the HIV-1 DNA PCR assay from 9 African countries with high-burdens of HIV/AIDS were shared with respect to different settings and HIV- subtypes. A consensus statement on the use of the assay for early infant diagnosis was developed and areas of needed operational research were identified. In addition, consensus was reached on the usefulness of dried blood spot (DBS) specimens in childhood as a means for ensuring greater accessibility to serologic and virologic HIV testing for the paediatric population.

## INTRODUCTION

Targets for the treatment of paediatric HIV infection are not being met in many African countries despite the increasing availability of the appropriate drugs needed for antiretroviral therapy (ART). African children infected with HIV are dying at an alarming rate. A meta-analysis published in 2004 showed that with no intervention approximately 35% of HIV infected children in Africa died before one year of age and that more than 52% died before their second birthday [1]. Data from Zambia demonstrated that 30-50% of infected infants died by two years of age [2] and recently published studies from South Africa found that 40% of HIV-infected infants died by 12 months of age [3, 4].****Additionally, these South African studies showed that over a 24 month period 60% of all infants were lost to follow-up by 6 weeks of age and 85% by 12 months, suggesting that one reason HIV-infected infants are not receiving timely access to ART is because most are not routinely accessing mother-child health (MCH) services early and HIV infection is not detected in a timely manner. It is critical to provide accurate and early infant diagnosis of HIV infection to ensure that comprehensive care and treatment including ART is offered to eligible infants, to evaluate prevention of mother-to-child transmission (PMTCT) programs, and to facilitate appropriate stratification of healthcare services [5].

Results of serologic HIV-1 diagnostic assays, the most widely used tools for diagnosing HIV infection, are difficult to interpret in infants and young children due to the persistence of maternal HIV antibodies for up to 18 months. As an alternative, the World Health Organization (WHO) and the United Nations International Children’s Emergency Fund (UNICEF) have recommended that countries provide access to virologic testing for HIV-exposed infants. To support national plans for scale-up of early infant diagnosis (EID) programs, high-burden countries are seeking guidance on choosing the appropriate technologies and testing algorithms using virologic or molecular assays. In the South African setting, it was shown that a single HIV DNA Polymerase Chain Reaction (PCR) test at 6 weeks of age was more useful to identify HIV infection among HIV exposed infants for early care and treatment than performing HIV enzyme immunosorbant assays (ELISA) at 12 months of age [1, 6]. However, factors in addition to cost, such as selection of who will be tested and when and where testing will be performed, have a direct impact on the choice of technology.

## METHODS

To address the need for scaling up HIV early infant diagnostic efforts the World Health Organization (WHO) and the U.S. Centers for Disease Control and Prevention (CDC) held a meeting in May 2006 in Entebbe, Uganda, attended by 60 participants from 17 countries. The goals of the meeting were to review the characteristics of available assays and share existing data on their performance in 9 African countries with high-burdens of HIV/AIDS including infections with different HIV subtypes; to discuss limitations of these assays in program settings, to identify areas where targeted operational research is needed to improve the performance of assays; and to develop consensus statements on the use of virologic assays for EID.

## RESULTS AND DISCUSSION

### Available Testing Methodologies for Early Infant Diagnosis of HIV Infection

A variety of commercially-available assays are currently in use including HIV DNA PCR [7]; HIV RNA assays [8-10] and the ultra-sensitive HIV p24 antigen assay [11] as well as in-house PCR assays that have been validated and are appropriately quality-assured. Although in-house, non-commercial, PCR assays can be validated according to the process recommended by the WHO [12], commercially available assays, while being more costly, offer the most suitable option for rapid scale-up of early infant diagnosis because they offer standardized reagents and adequate production and availability.

To address the utility of diagnostic technologies in early infant diagnosis in the meeting a variety of technologies were reviewed. The features of these technologies were summarized in Table **1**. These technologies include 1) Roche Amplicor HIV-1 Monitor™ and Manual Cobas Taqman [13]; 2) Abbott Real-Time HIV-1 [14]; 3) Bayer Versant^®^ HIV-1 RNA 3.0 [15]; 4) bioMerieux NucliSens^®^ HIV-1 QT and Nuclisens EasyQ HIV-1 [16]; 5) the qualitative Amplicor HIV DNA PCR v1.5 assay [7]; 6) Cavidi Exavir^®^ Load assay; and 7) the Perkin Elmer Ultra-sensitive HIV-1 p24 antigen assay [17].

The use of dried blood spot (DBS) specimens as the primary specimen for EID was an important topic for discussion during the meeting. DBS use represents a paradigm shift in accessibility to virologic testing for HIV infection. In addition to antibodies, nucleic acids in DBS have been shown to be stable for several months at ambient temperatures, provided the DBS specimens have been thoroughly dried and are stored with desiccant. Thus DBS specimens can be collected at remote rural sites and transported to a central or regional testing laboratory. However, specific training in the collection, labelling, drying and packaging of DBS is required to guarantee the quality of specimens for molecular analysis. In some countries with large-scale HIV counselling and testing programs, DBS specimens are used for routine quality control (QC) of rapid HIV serologic testing and thus healthcare staff have already been trained in the proper preparation and handling of DBS.

Supplies for DBS collection and shipment can be difficult to procure and several participants commented on the potential usefulness of a premade DBS sample collection kit containing all the necessary supplies for health care facilities. The precise contents of each kit for use in infant diagnosis or quality control of HIV testing would need to be determined for each country. Although other filter papers have been used for blood collection, Whatman 903 specimen collection paper (previously known as Schleicher and Schuell 903 paper) is the most widely used and validated paper for DBS collection.

### Role of HIV Antibody Assays in Children Less Than 18 Months of Age

**Fig. (1).Age-Stratified diagnostic pathways for well infants known to be HIV-exposed.**
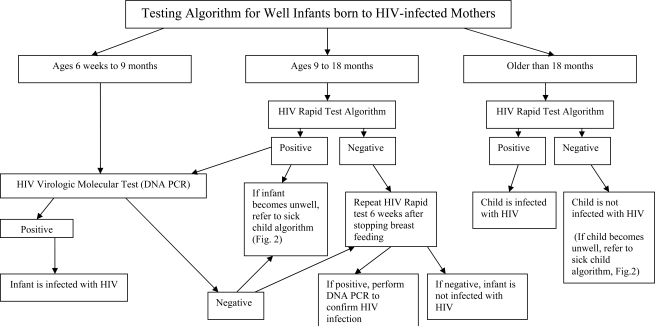
Different approaches are recommended for testing HIV-exposed infants based on age (35). Infants between ages 6 weeks and 9 months should not be diagnosed using an HIV Rapid test algorithm, but should be diagnosed by HIV DNA PCR. Children between 9 and 18 months should first be tested using an HIV Rapid test algorithm with positive tests confirmed by HIV DNA PCR (with immediate referral for care after a positive rapid test if there are long delays for the confirmatory test results). In children older 18 months of age, a positive result in the HIV Rapid test algorithm is indicative of HIV infection.

HIV antibody tests (either rapid tests or ELISA) are the preferred means of diagnosis of HIV infection in children older than 18 months of age. In contrast, a positive HIV antibody test at less than 18 months should not be used as the primary assay for EID because of the likelihood that the maternal antibodies might still be present. However, when performed according to robust national algorithms that accurately and reliably detect HIV antibody, a positive rapid test should trigger virologic or molecular testing. A study addressing the potential use of rapid HIV tests prior to molecular testing for early screening of pediatric HIV infection in Uganda was presented at the meeting [18]. The rationale behind this approach is that although a rapid test cannot establish that an infant is infected because the test cannot distinguish maternal from infant antibodies, it may be able to establish that the infant is uninfected since a close correlation had been observed between a negative rapid test result at any age and a negative test result by molecular assays. In the Uganda study, it was reported that the correlation was perfect in 138 healthy infants attending an immunization clinic, but at a pediatric AIDS clinic, only 49/57 (86%) of infants ranging in age from 6 weeks to 18 months were correctly identified as uninfected by an HIV-negative rapid test. Eight infected rapid test-negative infants had either a detectable viral load or HIV p24 antigen; and one of these infants was HIV antibody positive when re-tested using a different rapid test. A potential reason for these undetected infections is that rapid HIV testing may have been performed during the window period for the antibody response, thus implicating breastfeeding as the mode of transmission particularly in the older infants. These results indicate that further studies are needed to determine the age at which rapid HIV testing of exposed infants would be most cost-effective in relation to sensitivity, specificity, and positive and negative predictive values. However, the following issues suggest that introducing routine rapid HIV testing in the pediatric setting should be considered: 1) when compared to molecular testing, rapid tests are simpler, cheaper and more widely available; 2) a substantial proportion of uninfected, HIV-exposed children lose maternal antibodies before 9 months; 3) early identification and exclusion of HIV-uninfected infants could help focus limited HIV care resources on infected infants; and 4) breast-feeding infants can easily be retested by rapid tests 6 weeks after weaning. Based on evidence presented at the meeting and preliminary data from ongoing studies a consensus was reached at the meeting that if an HIV-exposed well child between 9 and 18 months of age had never been breast-fed or had stopped breast-feeding for at least 6 weeks and had a negative HIV rapid test result then the child should be considered uninfected with no further testing performed unless symptoms develop (Fig. **1**).

### Ultra-Sensitive HIV p24 Antigen Assay

The ultra-sensitive HIV p24 antigen assay is an ELISA, not a molecular assay, and is therefore suited to health facilities where serologic testing is routinely performed. The caveat to this is that few laboratories in Africa have been able to utilize the commercial Up24 antigen assay due to issues related to methodology and reagent supply. Nevertheless, the performance of the Up24 antigen assay for HIV diagnosis in infants and young children has been evaluated in a number of studies in countries with different HIV subtypes yielding sensitivities and specificities ranging from 96% to 99% [19-23]. Data from South Africa presented at the meeting confirmed that at 6 weeks the HIV p24 assay yielded satisfactory results with a sensitivity and specificity of 97.7% and 100% respectively when compared to HIV DNA PCR testing [11]. Recently Patton and colleagues confirmed the feasibility of using this assay on DBS, thus dramatically expanding its potential value in infant diagnosis [24]. This test uses washer and reader as used in a typical serologic laboratory and does not require thermocycler as required in PCR assays. The cost of the reagents is approximately 10-20 US dollars. A consensus was reached at the meeting that the measurement of HIV-1 p24 antigen in blood is sensitive enough for early diagnosis of HIV infection in infants and young children, but only if the ultra-sensitive HIV p24 antigen assay described by Schüpbach and colleagues is followed [25, 26].

### Total Nucleic Acid (TNA) Real-Time, Reverse-Transcriptase PCR Assay

TNA is a new approach specifically designed for use with infant DBS and, although still undergoing optimization and not commercially available, is being tested in Uganda. This semi-quantitative assay offers a number of potential benefits over commercial HIV DNA PCR assays including cost, increased sensitivity, increased through-put per run, less time to completion per run and ease of automation. An important feature of the TNA assay is the built-in internal control using a single copy human gene, RNaseP gene, to monitor the quality of the specimen and amplification process [27]. Data presented at the meeting from Uganda, where the prevalent HIV subtypes are A, C and D, suggested that the TNA assay performs with 100% sensitivity and specificity when compared to Amplicor HIV DNA PCR assay using 1800 DBS specimens from infants [28]. However, further studies are needed in other countries, and until the assay is available commercially, it can only be recommended as an ancillary assay or research tool in those laboratories with the necessary infrastructure and technological competence.

### HIV Amplicor DNA PCR v1.5 Assay

The Roche qualitative HIV Amplicor DNA PCR v1.5 assay provides an accurate method for identification of HIV-1 infection in infants and young children less than 18 months of age and is currently the only assay with extensive validation data in Africa and extensively used in the US and Europe [7]. Although designed for use with whole blood, the DNA PCR v1.5 assay can easily be adapted for use on DBS and this adaptation makes it particularly useful for early infant diagnosis in high-burden countries [30].

Early work from South Africa using DBS specimens collected from 300 infants at six weeks of age showed that the DNA PCR v1.5 assay performed exceptionally well with a sensitivity and specificity of 100% and 99.6% respectively, compared to the same assay conducted on fresh whole blood samples [30]. Preliminary data on 206 children suggested that DBS prepared from capillary (e.g. heel prick) versus venous blood also yields highly accurate results with a sensitivity of 98.3% and specificity of 98.7% (G. Sherman; personal communication). Participants at the meeting from Uganda, Zambia, Mozambique, Kenya, Namibia, Rwanda, Cote d’Ivoire, Botswana and South Africa shared data demonstrating the excellent performance of the DNA PCR v1.5 assay on DBS with respect to different settings and different HIV subtypes including perceived limitations of this assay in program settings.

Although the reports on the performance of the assay were generally positive, limitations to the assay were discussed during the meeting. First, although the assay has been shown to detect all HIV subtypes currently described, as with other molecular assays, it may not be capable of detecting future variants of HIV due to the virus’s ongoing evolution. Second, unlike the TNA real-time assay, there is no primer set that will amplify an internal control to ensure that DNA has been sufficiently extracted from each DBS specimen and is suitable for amplification. Third, as with any molecular test, there are issues related to staff expertise and training, instrument and space requirements, and cost.

Because comparison studies between DBS and whole blood/cell pellets using the DNA PCR v1.5 assay have been conducted in several countries, participants concluded that it is not necessary for each country to repeat these evaluations prior to implementation of DBS testing. Difficulties were described in testing DBS especially in high-volume laboratories since extraction of DNA from DBS is labour-intensive, prone to cross-contamination and may not always be efficient. Thus, it was recommended that DBS QC materials be included in each experimental run to ensure that the extraction procedure is effective. Strict criteria need to be established regarding acceptance of results for each experimental run, and specimens with low-positive results located in wells adjacent to specimens with strong-positive results should be routinely retested. Further work in automating DBS punching and DNA extraction is needed particularly for those laboratories testing large numbers of specimens.

### Internal Quality Control (QC) for HIV Amplicor DNA PCR v1.5

The importance of “Good Laboratory Practice” and adequate quality assurance for the DNA PCR v1.5 assay was strongly emphasized by all participants. As with all PCR-based technologies, the laboratory workflow should be designed to ensure that separate areas are provided for the performance of the assay to reduce the risk of cross-contamination. The need to harmonize standard operating procedures (SOPs) across the testing laboratory network was highlighted to include pre- and post-analytical phases of the process including sample collection, labelling and tracking, shipping and reporting.

A number of innovative approaches to monitor the overall performance of the Roche Amplicor DNA PCR v1.5 assay as part of internal QC were suggested including monitoring the number of internal positive control failures, the number of equivocal results and the number of false-positive and false-negative results as compared to a gold-standard. In Uganda, the Amplicor positive control failure was rare with this assay (1/975 specimens) and the equivocal result rate was low at 0.7%; false positive and false negative rates were 0.4% and 0% respectively [27]. Data presented from South Africa showed that between July 2005 and April 2006, 22,431 Roche Amplicor DNA PCR v1.5 assays were conducted with a combined internal positive control failure and equivocal result rate of 1-2% [31]. It was noted that this rate increased when new staff were introduced on the bench, when DBS technology was initially implemented, and when control specimen problems were identified, all of which highlight the need for continuous monitoring to identify possible laboratory staff who need re-training.

Participants at the meeting reported a variety of testing algorithms in use in their countries. In Uganda, specimens are extracted and tested in parallel on DNA PCR v1.5 and TNA; re-testing on an individual assay is triggered when internal quality control criteria are not met, and when results on both assays are valid but discordant, re-testing is performed on both assays. Using this algorithm, results with the two assays were 100% concordant after re-testing when necessary. In other laboratories where an alternative assay is not available, a specimen giving a positive result in the DNA PCR v1.5 is routinely re-extracted and re-tested. Consensus could not be reached at this meeting on the need to repeat a positive test result, even when the run was validated by all controls. This is probably a reflection on the fact that few laboratories have experience with DNA PCR v1.5 of more than a year and it will take some time before confidence in the results can be established. Meeting participants were in agreement that to ensure quality of testing during scale-up of programs, a subset of the positive DBS specimens should be tested at the national reference laboratory preferably with the same assay.

### External Quality Assurance (EQA) for HIV Amplicor DNA PCR v1.5

Most laboratories represented at the meeting were already implementing Amplicor DNA PCR v1.5 testing using DBS, suggesting an urgent need for an external quality assurance (EQA) program to monitor the quality of HIV PCR testing on DBS. To this end, the CDC-Atlanta International Laboratory Branch established an EQA program early in 2006. This proficiency testing (PT) program not only provides an external assessment of laboratory performance but also provides DBS specimens for internal quality control and technical assistance where needed. In the first round, ten DBS samples with varying HIV DNA content (0 to 100,000 copies/ml) were shipped to 17 participating laboratories from 11 different countries [32]. All laboratories participating in the program performed the DNA PCR v1.5 assay but used a variety of different DNA extraction methodologies including the Roche Manual Extraction, bioMerieux EasyMag and Roche MagNapure analysers. Of the 17 laboratories, 14 (83%) reported results that were in complete agreement with the expected results. There were no false positive results, and all false-negative results were from DBS specimens containing low copy number of HIV-1 DNA, suggesting inefficient extraction of DNA from the DBS. Similar difficulties with false-negative results on low-positive DBS specimens were seen in the second round of the PT program four months later when the number of participants increased to 22 laboratories in 13 countries (L.M. Parsons, personal communication). Technical support has been provided for the laboratories reporting false-negative results. It is recommended that all laboratories using the DNA PCR v1.5 for provision of early infant diagnostic services should enroll in a national or international EQA program.

### Large-Scale Implementation of the HIV Amplicor DNA PCR v1.5

As programs begin to scale-up, automation of the different steps in the HIV DNA PCR v1.5 assay will need to be introduced and evaluated. In the South African context where demand for HIV DNA PCR testing is high, automation of extraction has been evaluated using the Roche MagNapure Analyser. Overall, results to date have shown good correlation between the MagNapure extraction protocol and the Roche manual extraction procedure on cell pellets from whole blood specimens [7] but a slightly reduced sensitivity was seen when DBS specimens were used. The CDC-Atlanta International Laboratory Branch is evaluating automated extraction of nucleic acids from DBS with the Corbett X-tractor Gene instrument (Corbett Robotics, Queensland, Australia), and has found the instrument to provide rapid through-put with a sensitivity comparable to the Roche manual extraction method (R. Downing and C.-Y. Ou, personal communication).

There is an even more pressing need to automate the DBS punching step prior to analysis since the manual punching of discs is a rate-limiting step in the overall procedure and is highly prone to cross-contamination. Meeting participants indicated the need for further validation of a semi-automated punching instrument; the BSD 600-Duet Semi-Automated Dried Sample Punch Instrument (BSD Robotics, Queensland, Australia). With this instrument discs of 6 mm diameter can be punched directly into the Corbett 96 deep-well plates for automated extraction. The final step in the Roche DNA PCR v1.5 assay, the phase to detect colour development, is labour intensive and time-consuming and meeting participants indicated the need for the manufacturer to develop an automated version.

The overall design of a national EID program is dictated by the capacity of the testing laboratories and such capacity is dependent on the test adopted. To meet the high-volume testing needs in South Africa, testing is done by teams of technicians working in shifts, 24 hours each day. In Uganda where it is estimated that nearly 100,000 HIV-exposed infants in need of testing are born each year at least 12 testing facilities will be required, each with the capacity to perform 8,000 tests annually or 160 tests per week [33]. This is beyond the capacity of a single technician using manual punching and extraction protocols and reinforces the urgent need for research into various steps needed to optimize the assay.

### Opportunities for Early Infant Diagnosis Testing

Currently, routine follow up of HIV-exposed children and routine paediatric HIV counselling and testing (HCT) services are not standard in most African countries and the majority of HIV-infected infants are not identified until they develop symptoms and present illness at health care facilities. However, there are two opportunities for HIV-infected mothers and hence the exposed infants to be identified before symptoms occur. The first is at antenatal clinic (ANC) visits and the second is at postnatal immunization clinics. The first is either poorly attended or PMTCT services are not yet provided. In Uganda it is estimated that only 10% of ante-natal women have been tested and enrolled in PMTCT programs [18, 33]. In contrast, the infant immunization clinic visit at 6 weeks after delivery is generally well-attended and offers an opportunity to deliver HCT services, to identify infected mothers and exposed infants, and to refer them to care and treatment programs. The 6-week immunization clinic visit also coincides with the age at which the sensitivity of HIV DNA PCR assays peaks for those infants infected pre- or peri-natally. Further opportunities for testing exist at follow-up visits to the immunization clinic at 10 and 14 weeks after delivery, though these are less-well attended.

Linkages between ante-natal care and post-natal immunization clinics are generally weak or non-existent in many countries. At the meeting, data from Rwanda was presented on a pilot study designed to improve the linkages at three health centers in Kigali [34]. Antenatal women who had undergone HCT were requested to take their ANC card coded with their HIV status to routine immunization clinic visits where the mother’s card was stapled to the infant’s immunization card. By using the information from antenatal services, the HIV exposure status was known for 68% of children attending the three immunization clinics. Exposed infants were started on cotrimoxazole, offered HIV DNA PCR testing and referred for ART services if the test was positive. The immunization clinics also offered HCT services for those mothers whose HIV status was unknown and ensured that HIV-positive mothers received ART services if this had not been initiated during pregnancy. The routine infant immunization visits served as an entry point for HIV-infected and exposed children, and also as a ‘second chance’ to detect HIV-positive women not previously identified. The importance of strong linkages between ante-natal/post-natal clinics and ART programs for infected mothers and infants was mentioned by several participants.

### HIV Testing Algorithms for Infants and Young Children

**Fig. (2).Age-Stratified diagnostic pathways for sick infants where HIV exposure-status is unknown.**
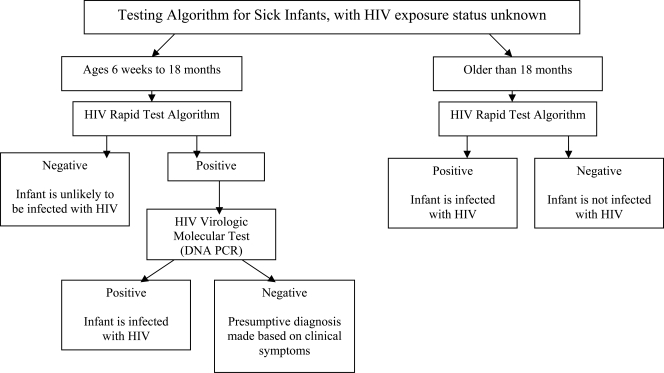
Different approaches are recommended for testing sick children based on age (34). All sick children should be tested first in an HIV Rapid test algorithm. A positive HIV Rapid test result in children under 18 months should be confirmed by HIV DNA PCR (with immediate referral for care after a positive rapid test if there are long delays for the confirmatory test results), while a positive HIV in children over 18 months of age is definitive of infection with HIV.

Testing algorithms, adapted from those recently proposed at the WHO meeting in Geneva in April 2006 [35] are shown for well children less than 18 months of age (Fig. **1**) and for sick children (Fig. **2**). Although there was general agreement on these testing algorithms at the meeting, there was some debate on the advisability of using a rapid HIV antibody test as a ‘screening test’ because some sick children with negative serology may in fact be positive using virologic or molecular assays (Fig. **2**) [18]. Further studies are needed to assess the sensitivity of rapid tests/algorithms against a gold-standard ELISA testing algorithm in infants and young children, particularly since use of rapid HIV tests offers the potential for faster referral and initiation of care. At the present time, WHO recommends that all children known to be exposed to HIV and all sick children suspected to be infected with HIV should be offered early virologic or molecular testing if resources and infrastructure allow.

### Recommendations for Laboratory Testing for the Diagnosis of HIV- Infected Infants and Young Children:

The Roche Amplicor DNA PCR version 1.5 assay has been validated in many countries. The assay is standardized, available in kit format, has the most widespread use, and is therefore recommended for EID programs. In addition, DBS is the preferred specimen type for two reasons: ease of collection from infants; and stability of DNA in the DBS during ambient temperature storage and shipment. Studies from several countries have demonstrated excellent correlation of results obtained using DBS and whole blood, and therefore it is not necessary for each country to repeat these evaluations prior to implementation of DBS testing.

The TNA assay is being used in some countries with excellent results. However, until the assay is standardized and available commercially, it can only be recommended as a confirmatory assay in those laboratories with the necessary infrastructure and technological competence.

The HIV p24 antigen assay is recommended for early infant diagnosis only if the ultra-sensitive protocol described by Schüpbach and colleagues is followed [26, 28], consistent supplies of test reagents could be obtained and technicians need to be well trained and certified to perform the assay.

The quantitative HIV RNA (viral load) assays are not currently recommended as diagnostic tools for the identification of HIV infection in infants, since they are expensive, technically challenging and have not been adequately evaluated using DBS specimens. Viral load assays are performed on whole blood specimens, and are primarily used along with CD4 counts for assessing disease progression, patient care and treatment management. However, a molecular laboratory in Cote d’Ivoire has started using viral load analysis on whole blood samples for EID testing in spite of challenges encountered with sample collection and transportation from the clinical sites to the testing laboratory. While this laboratory has overcome these challenges, scale-up of EID service to remote geographic areas would not be practical until a careful evaluation and validation of viral load testing on DBS specimens is performed.

Before initiating EID programs, written national SOPs need to be developed to include pre- and post-analytical phases such as sample collection and tracking, shipping and reporting as well as the analytical phase. These SOPs should be widely available and staff should receive specific training in the collection, labelling, drying and packaging of DBS to guarantee the quality of specimens. Supplies for DBS collection, packaging and shipment should be available in kit form from national procurement and distribution centers.

DBS specimen-rejection criteria should be established and if more than 2% of DBS from a specific health facility are rejected, this should trigger re-training of the staff responsible for specimen collection, storage and transport. Internal quality assurance should include monitoring the number of internal positive control failures, the number of equivocal results and the number of false-positive and false-negative results as compared to a gold-standard. Overall competency of testing laboratories should be continuously monitored using proficiency testing panels (EQA) and by QC re-testing at the national referral laboratory. The overall performance of the laboratories supporting the national EIDP should be monitored by a central coordination unit within the Ministry of Health that will provide support supervision, and to which all participating laboratories should submit regular reports.

## ONGOING OPERATIONAL RESEARCH STUDIES

### Molecular Testing

The Roche HIV-1 DNA PCR assay is labour-intensive and time consuming. Several approaches are currently being evaluated for automation of different steps in this assay. Commercially-available HIV-1 RNA assays used for ART monitoring should be evaluated for possible use in the diagnosis of HIV infection in infants and young children, preferably using DBS specimens.

### Serology

It would be advantageous for the management of EID programs to exclude HIV-uninfected children earlier than 12 to 18 months with a simpler and less expensive rapid test. Research to determine the mean age at which sero-reversion (disappearance of maternal antibody) occurs using rapid tests would greatly facilitate early identification of uninfected children, perhaps at nine months of age or earlier. If pre-screening with rapid tests at an earlier age is found to be useful, the most appropriate rapid tests/algorithms need to be identified.

### Combination Algorithms

Studies are also needed to determine if results of rapid HIV serologic testing in combination with CD4+ T-cell count and a clinical assessment would offer a timely, cost-effective alternative to the current algorithm of virologic/molecular testing with or without CD4 count for identifying infants eligible for ART. In addition, programmatic studies should evaluate the cost-effectiveness of various algorithms, particularly cost differences associated with the timing of testing.

## CONCLUSIONS

Accurate laboratory diagnosis of HIV-1 infection in infants is necessary to initiate essential treatment, however use of appropriate and reliable laboratory testing is challenging in countries with high burdens of disease [36]. Recent reports suggest that the Amplicor HIV DNA PCR version 1.5 assay represents the best option currently available for immediate scale-up of EID programs in the majority of African settings [2, 18, 31, 33, 34]. This assay appears to have acceptable sensitivity for the detection of the common HIV-1 subtypes. Ongoing molecular surveillance is advised due to the diversity of HIV and its continuing evolution to ensure that molecular assays remain relevant within local populations. Continued evaluation of new technologies as they arise is recommended as well as significant operational research to ensure that the most up-to-date diagnostic algorithms are used. Finally, programmatic aspects have to be carefully considered to assure the widest possible access to HIV services for mothers and children.

## Figures and Tables

**Table 1:** Commercial Assays Can Potentially be Used for Diagnosis of HIV Infection in Infants and Young Children

Source/Name of Assay	Sample Used	Assay/Target	Volume (in ml)	Cost Per Test ($)
Roche	Whole Blood	Qual/DNA	0.5	35- 90
Amplicor HIV-1				
DNA v1.5				
Roche	Plasma or serum	Quan/RNA	0.2-0.5	20-50
Amplicor HIV-1 Monitor v 1.5 manual				
RocheCobas Amplicor HIV-1 Monitor v 1.5	Plasma or serum	Quan/RNA	0.2-0.75	20-50
Roche	Plasma or serum	Quan/RNA	0.5-1	50
TagMan 48				
Bayer	Plasma or serum	Quan/RNA	1	85-90
Versant				
HIV-1 RNA				
Biomerieux NucleiSens EasyQ	Plasma or serum	Quan/RNA	1	80
Abbott	Plasma or serum	Quan/RNA	0.2-1	20-70
Real Time HIV				
CavidiExaVir Load	Plasma or serum	Quan/		1	13-15
		RT			
Perkin Elmer	Plasma or serum	Quan/	50 µl	10
Up24 antigen		p24 antigen		

*Qual- Qualitative and **Quan- Quantitative.
